# High-Frequency Ultrasound Dataset for Deep Learning-Based Image Quality Assessment

**DOI:** 10.3390/s22041478

**Published:** 2022-02-14

**Authors:** Joanna Czajkowska, Jan Juszczyk, Laura Piejko, Małgorzata Glenc-Ambroży

**Affiliations:** 1 Faculty of Biomedical Engineering, Silesian University of Technology, Roosevelta 40, 41-800 Zabrze, Poland; jan.juszczyk@polsl.pl; 2 Institute of Physiotherapy and Health Sciences, Jerzy Kukuczka Academy of Physical Education, Mikołowska 72a, 40-065 Katowice, Poland; l.piejko@awf.katowice.pl; 3 Amber Academy, Piownik 3, 44-200 Rybnik, Poland; amber.szkolenia@gmail.com

**Keywords:** high-frequency ultrasound, image classification, deep learning, transfer learning, image quality assessment

## Abstract

This study aims at high-frequency ultrasound image quality assessment for computer-aided diagnosis of skin. In recent decades, high-frequency ultrasound imaging opened up new opportunities in dermatology, utilizing the most recent deep learning-based algorithms for automated image analysis. An individual dermatological examination contains either a single image, a couple of pictures, or an image series acquired during the probe movement. The estimated skin parameters might depend on the probe position, orientation, or acquisition setup. Consequently, the more images analyzed, the more precise the obtained measurements. Therefore, for the automated measurements, the best choice is to acquire the image series and then analyze its parameters statistically. However, besides the correctly received images, the resulting series contains plenty of non-informative data: Images with different artifacts, noise, or the images acquired for the time stamp when the ultrasound probe has no contact with the patient skin. All of them influence further analysis, leading to misclassification or incorrect image segmentation. Therefore, an automated image selection step is crucial. To meet this need, we collected and shared 17,425 high-frequency images of the facial skin from 516 measurements of 44 patients. Two experts annotated each image as correct or not. The proposed framework utilizes a deep convolutional neural network followed by a fuzzy reasoning system to assess the acquired data’s quality automatically. Different approaches to binary and multi-class image analysis, based on the VGG-16 model, were developed and compared. The best classification results reach 91.7% accuracy for the first, and 82.3% for the second analysis, respectively.

## 1. Introduction

During the last decades, high-frequency ultrasound (HFUS, >20 MHz) has opened up new diagnostic paths in skin analysis, enabling visualization and diagnosis of superficial structures [1,2]. Therefore, it has gained popularity in various areas of medical diagnostics [3,4] and is now commonly used in medical practice [5]. In oncology, it helps in the determination of skin tumor depth, prognosis, and surgical planning [1,6], enabling differentiation between melanoma, benign nevi, and seborrheic keratoses [7]. Heibel et al. [6] presented the HFUS as a reliable method with perfect intrahand interreproducibility for the measurement of melanoma depth in vivo. Sciolla et al. [8] described the spatial extent of basal-cell carcinoma (BCC), provided by HFUS data analysis, as a crucial parameter for the surgical excision. Hurnakova et al. [9] investigated the ability of HFUS (22 MHz) in rheumatology to assess cartilage damage in small joints of the hands in patients with rheumatoid arthritis (RA) and osteoarthritis (OA). In the newest study, Ciapoletta et al. [10] describe the usefulness of using 22 MHz ultrasound images for hyaline cartilage diagnostics. The skin thickness and stiffness measurements are recognized by Chen et al. [11] as a valuable supplement to clinical skin assessment in systemic sclerosis. In dermatology, HFUS images are most applicable for a subepidermal low echogenic band (SLEB) below the echo entry (epidermis layer) detection, which may indicate inflammatory skin disease [12], and its thickness correlates to the severity of this lesion [2,12]. In patients with atopic dermatitis (AD), apart from diagnosis support, the HFUS is also useful for epidermal atrophy monitoring in topical corticosteroid treatment. Thanks to the reliable, accurate, and fast skin layer visualization, including epidermis, dermis, subcutaneous fat layer, the muscle layer, blood vessels, and hair follicles, the HFUS found applications in aesthetic medicine. Recently, Levy et al. [3] reported its usability for healthy skin analysis, where the increased collagen production connected with aging causes the skin echogenicity to increase.

Usually, the development of imaging techniques is followed by the fast development of dedicated image processing algorithms. In recent years, there appeared in literature [2,4,10,13,14,15] different solutions in computer-aided diagnosis (CAD) of skin in HFUS data, which target segmentation, detection, and classification of the affected areas. A robust skin layer segmentation in HFUS images was as first described by Gao et al. [16], and developed by Sciolla et al. [15], to finally gain Dice index of 0.919 in [17] for epidermis segmentation, and 0.934 for fetus body segmentation in embryonic mice HFUS volume image analysis. The skin tumor segmentation frameworks in HFUS data start from [8] to finally reach Dice of 0.86 for skin tumor segmentation in clinical dataset [18]. An extension to this targeting skin layer segmentation for accurate tumor seed point detection is described in [14]. The first solution of HFUS image classification is described in [4], where the considered cases include inflammatory skin diseases, skin tumors, and control group. All the mentioned techniques assume that the preselected correctly acquired images are proceeded and do not evaluate the incorrect input data. A completely different view of the classifications problem is presented in [10], where the authors divide the acquired HFUS records of rheumatic diseases into sets of informative and non-informative frames. The US frame was defined as informative when it shows enough information to fulfill the Outcome Measure in Rheumatology US definition of healthy hyaline cartilage.

Since the development of HFUS image analysis coincided with the dynamic development of machine learning algorithms, especially in the area of deep learning, most of the newest approaches [4,10,17] utilize their benefits. The first applications of convolutional neural network (CNN) to HFUS image segmentation are described in [2,19]. The U-shaped network, extending the conventional U-Net architecture by batch normalization layers, accurately segmented epidermis and SLEB layers. The same architecture, followed by the Savitzky–Golay filter and Fourier Domain Filtering, is described in [20] for epidermis and hair follicle segmentation in optical coherence tomography images. A development of [2] is the framework described in [17], where the authors expanded the CNN-based approach by fuzzy connectedness analysis for robust epidermis segmentation in HFUS.

The most common application of deep learning in medicine is data classification. Huang [21] and Cai [22] described its usage in ultrasound (US) for breast diagnosis support. Huang et al. [21] broadened this scope by liver, fetal and cardiovascular image classification, and thyroid nodule diagnosis assessment. Next, the list was extended by Liu et al. [23], who added kidney, bone, prostate, and brain US images. There are different architectures generally utilized in US data classification, like GoogLeNet, for breast lesions [24] and thyroid nodules [25]; VGGNet and fully-connected network (FCN), which face the level of liver fibrosis differentiation problem [26]; or Inception-v3, ResNet-101, and DenseNet-169, achieving the best performance in automatic classification of common maternal–fetal ultrasound planes [27].

The problem which arises with the development of CNN is the access to a large amount of labeled data. To fill this gap, the authors and institutions increasingly publish the data sets through Mendeley Data [28], Center for Artificial Intelligence in Medicine and Imaging [29], GitHub, or other repositories. However, these repositories leave much to be desired for the newest imaging techniques, and only one dataset of HFUS skin images, described in in [30], can be found in Mendeley Data. We collected and shared the face HFUS image database described in this paper to meet this need.

One of the possible solutions, which can partially solve the overfitting problem, if training from scratch, is data augmentation. Nevertheless, a feasible alternative is to use: Semi-supervised learning, transfer learning (TL), learning from noisy labels, or learning from computer-generated labels [31]. However, TL is reported as widely applicable in medical image processing tasks [32,33], where limited training data are common problems. In this approach, the knowledge is extracted from well-annotated, available, large datasets (e.g., ImageNet [34]) and used in the ongoing issues.

Fast and robust classification steps in medical applications are essential for further clinical practice usage. Moreover, the visual explanation of the system decision (like Grad-CAM map [35]) enables its recommendation for clinical use (‘explainable AI’). Noise or the artifacts influencing the geometry of visualized structures may lead to misclassification, false-positive detections, over/under segmentation, and in consequence, inaccurate results of measurements. To solve these problems, image quality assessment (IQA) algorithms are developed [36,37,38]. Very popular yet poorly correlating with human judgments of image quality are mean-squared error (MSE), its relevant peak signal-to-noise ratio (PSNR), or a bit more efficient structural similarity index (SSIM) [39]. All the mentioned assume that the original image signal is known. According to [40], the optical images can be distorted at any stage of their acquisition, processing, compression, etc., and a reliable IQA metrics is critical for evaluating them. The distortion-specific BIQA (blind image quality assessment) methods provide high accuracy and robustness for known distortion types or processes. Unlike the previous methods, these do not require the original image availability. However, considering that the distortion type is specified quite rarely, their application scope is limited. Therefore, natural scene statistics (NSS), including local DCT (discrete cosine transform) or wavelet coefficients describing contrast or gradient features, are utilized [41]. The DGR (distortion graph representation) based solution is presented in [40]. It considers the relationship between distortion-related factors and their effects on perceptual quality. Since the blind measures are distortion-specific, the blind no-reference (NR) IQA methods have been studied in recent years [39]. Both the BIQA and NRIQA are extended to work with stereo images [42], VR images [43], and many other currently investigated image types. As reported in [37], most IQA methods and research studies focus on optical images. Since the medical image quality is highly related to its application, and in some issues, low contrast and noisy images can still be acceptable for medical diagnosis, medical image quality assessment differ from the others [36]. They consider multiple expert opinions to label the data and utilize the benefits of AI (artificial intelligence). The applications of CNN to IQA of retina images can be found in [38]. The authors use DenseNet to classify the images into good and bad quality or five categories: Adequate, just noticeable blur, inappropriate illumination, incomplete optic disc, and opacity. Piccini et al. [44] utilized the benefits of using CNN to assess the image quality of whole-heart MRI. The only two solutions for ultrasound IQA, both based on CNN, are given in [37,45]. The chronologically first [45] scheme targets to assess the fetal US image quality in the clinical obstetric examination. The second one [37] is claimed to be universal, considering different US images. In the designed framework, the network is trained on the benchmark dataset LIVE IQ [46] and then fine-tuned using ultrasound images.

As we mentioned before, the HFUS image processing algorithms described in the literature [2,14,15,17] assume that the input dataset consists of preselected good quality image data. Among many possible applications, CNNs are for the first applied to reduce the analyzed dataset of HFUS to the informative part in [10]. In the current work, we decided to follow this way and automatically select the correct frames from the acquired dataset—asses the HFUS image quality. This solution enables automated analysis of HFUS records, which avoids the influence of incorrect detections on the analysis results. Due to the absence of such frameworks for HFUS skin images, the two main contributions of our study are as follows. The first is the database, including 17,425 HFUS frames of facial skin denoted by two experts (in total three times) as noisy-inaccurate for analysis and correctly acquired [47]. The proportion of correct and incorrect data is about 1:1.3. The data description includes the demographic features of the patient cohort, places of image acquisition on the face, acquisition dates, and system parameters. Second, we present different deep learning-based frameworks, including followed by a fuzzy interference system for automatically annotating frames. The analysis is conducted two-way, classifying the data into correct and incorrect and dividing them into four groups, depending on the experts’ majority decision.

Our extensive image database includes data acquired during an actual dermatological ultrasound examination. Thus it contains:

- images distorted by artifacts from trembling hand with the US probe or impurities contained in the ultrasound gel;
- frames captured when the ultrasound probe was not adhered or incorrectly adhered to the patient’s skin, or the angle between the ultrasound probe and the skin was too small (the proper angle is crucial for HFUS image acquisition);
- images with too low contrast for reliable diagnosis, or captured with too little gel volume-improper for epidermis layer detection;
- data with disturbed geometry as well as HFUS frames with common ultrasound artifacts like acoustic enhancement, acoustic shadowing, beam width artifact, etc.

**Figure 1:**
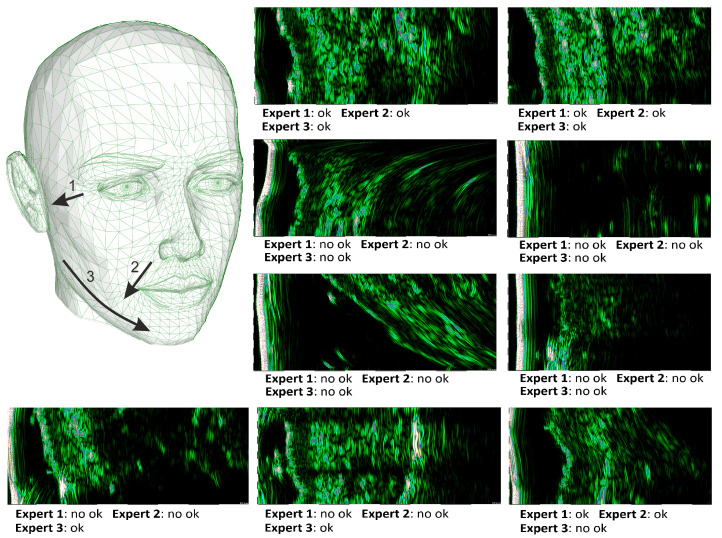
Facial model with superimposed image acquisition areas, and exemplary HFUS images, annotated by the experts.

Due to the image variety, high amount of possible distortions, and the subjective expert opinion, which is not always connected with them, application of IQA methods dedicated to optical images is impossible (Zhang et al. underline it strongly in [37]). A portion of images are hard to decide (even by the experts, see Figure 1), they can be useful in the diagnosis, but due to some artifacts, their analysis might be error-prone. Therefore, following the works in medical IQA [37,38,44] and image selection [10], we propose the CNN-based framework-a combination of the previous, which enables HFUS skin image analysis. The images selected by our methods are high quality, or informative, and accurate for diagnosis and processing. Depending on the application and user needs, the obtained results can be utilized twofold. First, only those classified as definitely good for the high amount of the acquired frames (label 4 in Table 1) should be considered. Second, for the US record with a limited number of frames, the image data labeled as 2 and 3 (in Table 1) can be taken into account. Yet, the results of their further automated analysis (segmentation or classification) should be treated as less trustworthy (assuming two trustful levels: Higher and lower, connected with labels 2 and 3, respectively). This is the first application of CNN to this task in HFUS images and the first combining CNN and fuzzy interference system.

The dataset developed in this study is in detail described in Section 2. The description is followed by numerical analysis of the data and expert annotations. The classification steps are presented in Section 3, including two- (Section 3.1) and multi-class Section 3.2 analysis. The model assessment and other results are given in Section 4. The study is discussed and concluded in Section 5.

## 2. Materials

The dataset includes high-frequency images (image sequences) of female facial skin. The data were collected during 4 sessions (the session dates are given as data IDs in a format [day month year]), with 44 healthy Caucasian subjects in age between 56 and 67 years (average = 60.64, std = 2.61), all postmenopausal. In anti-aging skin therapy, the patients were treated with trichloroacetic acid (TCA) chemical peel. The first image data were acquired before the first acid application, and the patients were divided into treated (23), and placebo group (21). The data were registered from three different locations on the patient face. The locations and ultrasound probe movement directions are visualized in Figure 1 by three arrows superimposed into a facial model. The image acquisition starts where the arrow begins and ends with the arrow end. At each patient visit, three HFUS series were registered. Several dozen (about 40) HFUS images were collected in a single series for each location. The original image resolution was equal 1386×3466 [pix] and the pixel size is equal to 0.0093×0.0023 [mm/pix] (axial × lateral). The analyzed HFUS image data were acquired using DUB SkinScanner75 with a 24 MHz (B-mode frequency, 8 mm depth, and acoustic intensity level 40 dB) transducer. Each series includes both the image data suitable for further diagnosis (technical-using CAD software, or medical) or not. The second group includes, for example, the ultrasound frames captured when the ultrasound probe was not adhered or incorrectly adhered to the patient’s skin and when the angle between the ultrasound probe and the skin was <70 degrees. Exemplary HFUS images annotated as suitable (‘ok’) or not (‘no ok’) for further analysis are given in Figure 1.

The HFUS examinations were performed by a beginner sonographer (without any experience in HFUS image acquisition and analysis, but working with the conventional US in his scientific practice): ID = 15022021 and 12042021, and experienced one (graduating Euroson School Sono-Derm, and working with HFUS image analysis from 3 years): ID = 08032021 and 07062021. In total 17,425 HFUS images were acquired.

**Figure 2:**
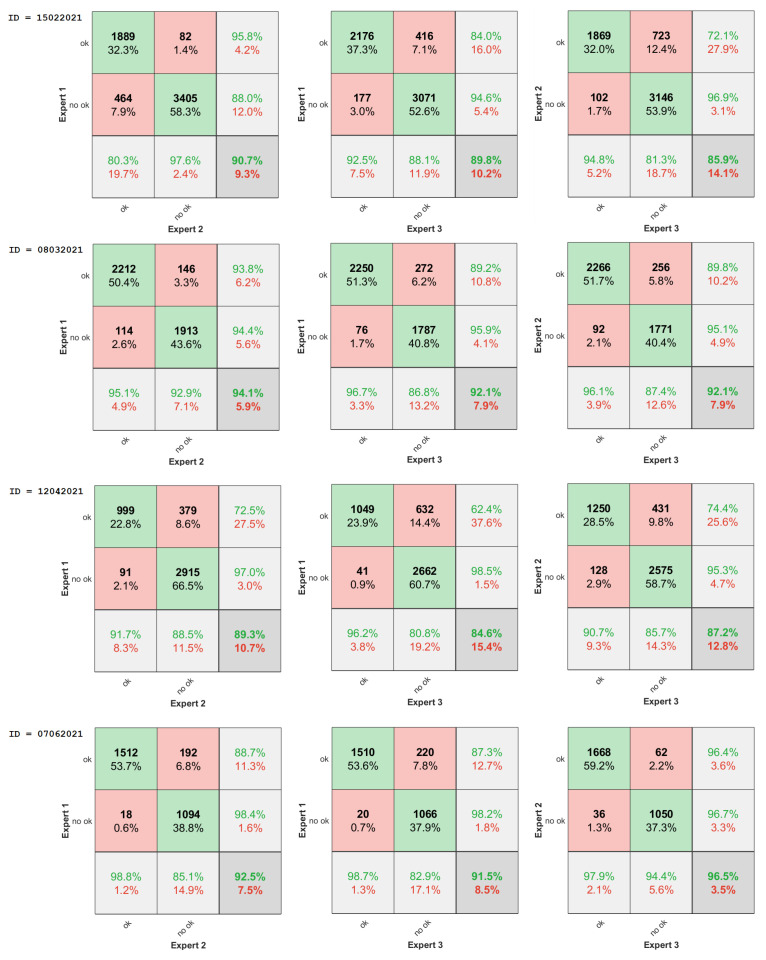
Inter/intra-observer agreement-confusion matrices.

**Figure 3:**
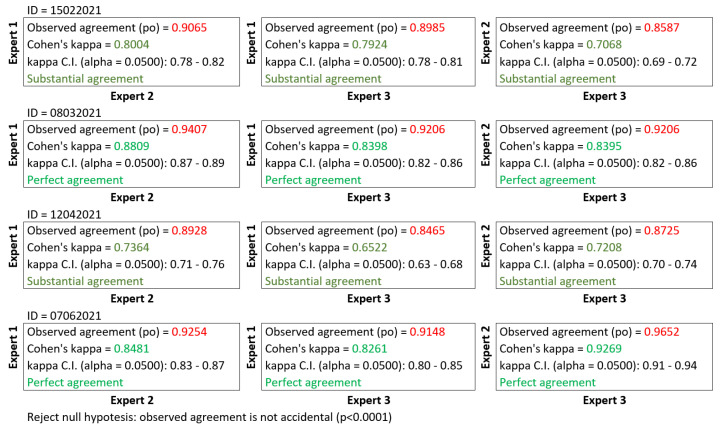
Inter/intra-observer agreement—unweighted Cohen’s Kappa.

After the data collection step, the complete dataset was labeled by **two experts** in HFUS data analysis. One of them annotated the data twice with an interval of one week. Hence, the further description includes **three annotations** denoted as **Expert 1**, **Expert 2**, and **Expert 3**. However, the labels **Expert 1** and **Expert 2** refer to the same person (annotations of the first expert with a week interval). The agreement in the useful image selection between all the experts was analyzed statistically using both confusion matrices (given in Figure 2) and unweighted Cohen’s Kappa [10], and interpreted according to Cipoletta et al. [10], and Landis and Koch [48] (see Figure 3). The analysis was performed using Matlab library [49]. The agreement between experts was partially substantial or perfect, and there is no difference between intra- and inter-observer results.

For further analysis, based on the expert majority decision, the data were additionally classified into four groups: 1—All experts labeled the image ‘no ok’; 2—one expert tagged the image ‘no ok’; 3—two experts labeled the image ‘no ok’; 4—all experts labeled the image ‘ok’. The size of individual groups (number of images) is collected in Table 1. Considering groups 1 and 4 only, the proportion of correct (’ok’) and incorrect data (‘no ok’) is about 1:1.3, and it increases to 1:2 in case of examination performed by a beginner sonographer. The most significant difference between these two sonographers considering the expert labels is the proportion of 4th labeled data to the total number of registered scans. In the case of the experienced sonographer, it equals 50%, and for the inexperienced one, only 27%.

**Figure 4:**
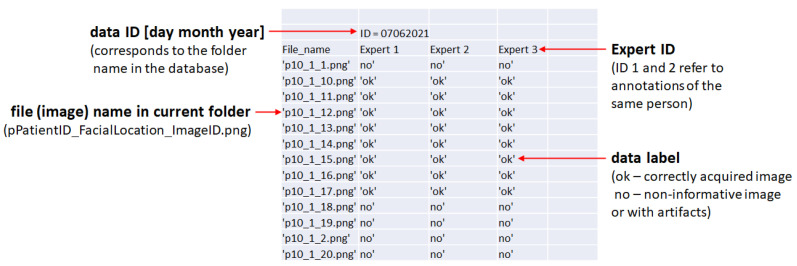
ID_DataDesc.xls file structure.

The data are publicly available under [47]. The consecutive examinations are collected in folders. The folder names correspond with the data IDs. Expert annotations of each folder are provided in .xls files denoted as ID_DataDesc.xls. ID_DataDesc.xls files structure is shown in Figure 4. The File_names are coded as follows: ‘**p**PatientID**_**FacialLocation**_**ImageID**.png**’. The database information is listed in Table 2. The dataset can be used as a benchmark for HFUS image classification, analyzed with the provided pre-trained CNN models, or utilized for other applications, like skin layer segmentation (for this, additional expert delineation is required). Due to the limited space, the image data are provided in the size of 224 × 224 × 3. The sizes result from the pre-trained CNN models input, as described in the further part of the paper. Readers interested in the image data in original size, please contact the corresponding author. The data repository [47] contains the trained CNN models described in this work, as well as the fuzzy interference system providing the final classification results.

## 3. Methods

There are different ways for ultrasound-based diagnostic procedures. Depending on the application, the sonographer acquires either a single image or an image series. The second approach is better when a further automated image processing step is introduced. Simultaneous analysis of multiple data provides reliable results, less prone to artifacts and outliers. At the same time, the analysis of the whole recording might be disturbed by strongly distorted data or the artifacts influencing the geometry of visualized structures, appearing on the part of frames. Consequently, it leads to misclassification, false-positive detections, and finally, inaccurate results of measurements. Therefore, the overall goal of this study was to develop and evaluate the classification framework, which enables robust and fast HFUS series analysis.

Numerical analysis of image annotations provided by the experts, described in Section 2 shows that manual image labeling is a nontrivial issue. While most of the images were unambiguously annotated as correct or not, there appear image data (in our case, it is 15%) on which the experts disagree. There are images partially disturbed in this group but still having diagnostic potential. Considering this, we first divide the data into unambiguous and ambiguous. It enables CNN model selection, suitable for further analysis. Then, the developed methods followed twofold: Binary classification and multi-class analysis. The first one includes division the image data, and two groups are denoted as correct and incorrect. Next, the data will be divided into two and four groups, respectively, according to the labels included in Table 1.

### 3.1. Binary Classification

The first goal of this step is the CNN model selection, providing the most reliable classification results. Based on the previous experiences [4] and the recent papers in medical IQA [38], or informative HFUS image selection [10], we consider two most promising architectures. The first one is DenseNet-201 [50] and the second is VGG16 [51]. Both were pre-trained on the ImageNet dataset and then used for transfer learning. DenseNet uses features of all complexity levels, giving smooth decision boundaries and performing well when training data is insufficient, whereas VGG16 is described as being suitable for the small-size training set and low image variability [10]. Both architectures were adapted for the binary classification problem. The DenseNet-201 architecture was trained freezing the first 140 convolution layers (as in [4]) and tuning the remaining ones, whereas in the VGG16 model, according to [10], 10 convolution layers were frozen.

Prior training, RGB US frames were resized to 224 × 224 × 3 pixels. The stochastic gradient descent optimizer with the momentum of 0.9, the categorical cross-entropy as loss function, batch size equal to 64, and initial learning rate of 0.0001 were chosen as the most efficient in a series of experiments [4,10]. The authors of [10] suggested 100 epochs for training the VGG16 model. However, due to the observed overfitting problem (the validation accuracy does not change, but the validation loss increases), we shortened the training process to 50 epochs. In further iterations, no significant improvements in training curves were visible, and the validation loss tended to increase. The same training parameters were applied for binary and multi-class models.

**Figure 5:**
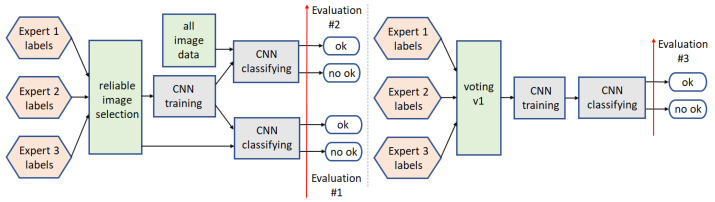
Binary classification schemes: Path1 (**left**) and path2 (**right**).

**Figure 6:**
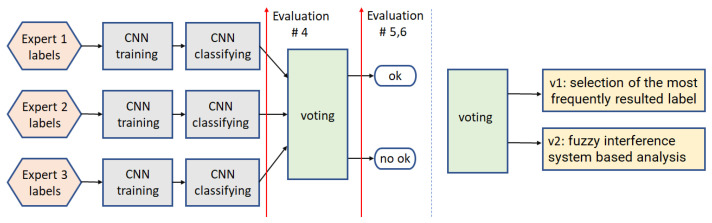
Binary classification schemes: Path3 and path4, utilizing two different voting algorithms, v1 and v2, respectively.

For the binary classification, the models are trained five times (see annotations ‘CNN training’ in Figure 5 and Figure 6). Three of them are connected with three separate expert annotations (Expert 1 labels, Expert 2 labels, Expert 3 labels). The fourth one considers only the part of the data on which the experts agreed (labels 1 and 4). In contrast, the fifth one (in path2) utilizes the labels resulting from the previous voting step—selecting the most frequently indicated label. This models are utilized in four processing paths shown in Figure 5 and Figure 6, and described below.

The voting step utilized in binary classification targets is calculating a binary output based on three labels provided by the experts or resulting from the analysis. The first solution is applied in path2, where the binary labels required for model training are calculated based on the expert annotations. The US frame indicated two times as ‘ok’ is considered as ‘ok’, and the US frame indicated twice as ‘no ok’ is considered as ‘no ok’. It corresponds to Group labels (in Table 1): 4 and 2 for ‘ok’, and 1 and 3 for ‘no ok’, respectively. In path2, three separate models (one for each expert) are trained and tested, and the final binary classification results are calculated as previous: The label resulting twice determines the output. The binary output selection used in path4 is described in detail in Section 3.1.4.

#### 3.1.1. Path1

This scheme (Figure 5 left) starts from the reliable images selection step, based on annotations provided by all the experts. By reliable images, we understand this part of the input data, for which all the experts agreed (labels: 1 and 4 from Table 1). The CNN model is trained and then applied to all the image data (labels 1 to 4).

#### 3.1.2. Path2

In this processing path (Figure 5 right), the CNN model is trained based on all the input data, and the binary input labels are calculated based on the voting step (v1). The voting step (v1) selects the most frequently indicated label, among three experts annotations.

#### 3.1.3. Path3

This framework (Figure 6, v1) is based on the CNN training and then classifying, performed for each independent expert input. The obtained results are then used for voting (v1)—selecting the most frequently resulting label.

#### 3.1.4. Path4

**Figure 7:**
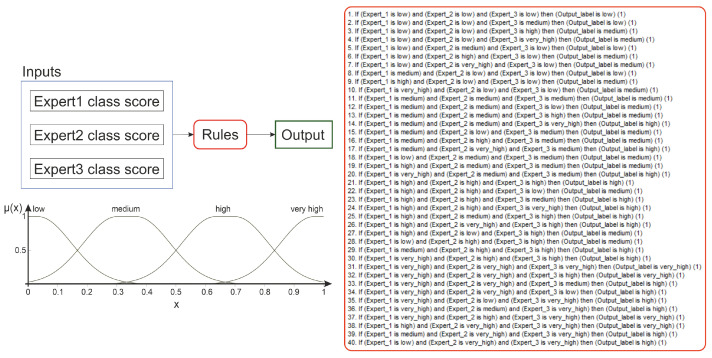
Fuzzy Interference System.

This path (Figure 6, v2) refers to the same framework as path3 with the difference that the voting step utilizes Mamdani Fuzzy Interference System (FIS) [52], followed by uniform output thresholding (t∈{0.25,0.5,0.75}) for final decision (see Figure 7). The membership function of the fuzzy sets in the rule premises and conclusions look the same for inputs and output and are presented in Figure 7. As the FIS input, we introduce the CNN predicted class scores. The FIS output can also be used as the confidence measure for further analysis, where the images classified as ‘definitely’ correct are rewarded.

### 3.2. Multi-Class Analysis

**Figure 8:**
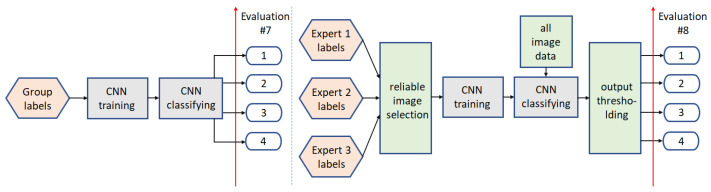
Multi-class analysis: Path5 (**left**) and path6 (**right**).

**Figure 9:**
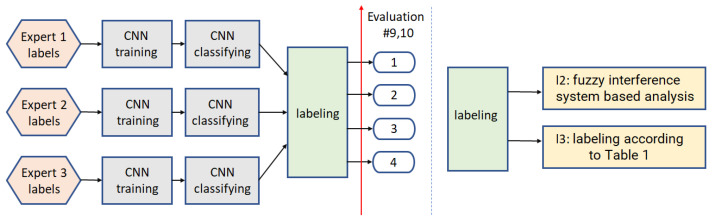
Multi-class analysis: Path7 and path8, utilizing two different labeling algorithms, I2 and I3, respectively.

The multi-class analysis is performed twofold. In the first solution, the previously obtained binary classification results are combined to provide the final results. In the second one, the model is adapted to 4-group classification and trained again. Same as before, different processing paths are introduced to obtain the final classification results (see Figure 8 and Figure 9).

#### 3.2.1. Path5

In the first experiment, the Group labels defined in Table 1 are used for 4-group CNN model training. The trained model is then directly used for data classification.

#### 3.2.2. Path6

The second processing path here (path6) refers to path1 in binary classification. The CNN model is trained on the reliable image data, then used for all data classification, and the predicted class score is uniformly thresholded to obtain the final classification results.

#### 3.2.3. Path7

Path7 refers to path3 in binary classification. Three CNN models are trained separately, and the final labeling is based on the scheme given in Table 1, with the difference that we do not take into account the expert annotations, but the results of the three models.

#### 3.2.4. Path8

The final proposed approach, same as path4, introduces Mamdani FIS with uniform output thresholding (t∈{0.25,0.5,0.75}). The final group labels are calculated as follows: FIS Output <0.25-label 1, FIS Output ∈[0.25,0.5]-label 3, FIS Output ∈[0.5,0.75]-label 2, FIS Output >0.75-label 4. The system differs in the Rules set, and both of the FIS systems (for path4 and path8) are provided in [47] as FIS2.fis and FIS4.fis, respectively.

## 4. Experiments and Results

**Figure 10:**
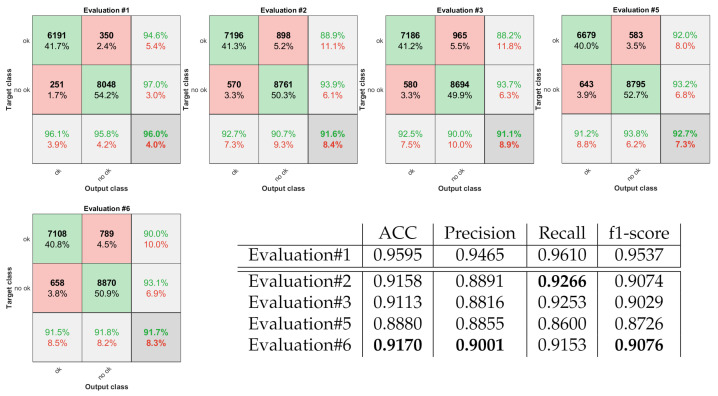
Confusion matrices and classification performance measures obtained for binary classification.

**Figure 11:**
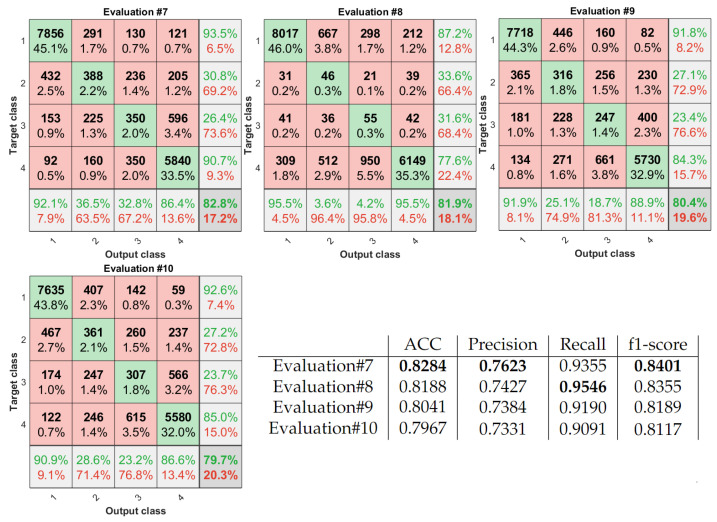
Confusion matrices and classification performance measures obtained for multi-class analysis.

To assess all the experiments, we used the external 5-fold cross-validation, and the non-testing remaining data were divided into training and validation subsets (4:1 ratio). All the experiments are marked on the classification schemes using red arrows and ’Evaluation #nb’ tags. To measure the performance of all the introduced approaches, we compute the accuracy (ACC), the classification Precision, Recall, and f1-score. Additionally, due to the class imbalance, we use confusion matrices to capture all the classified and misclassified records classwise (see Figure 10 and Figure 11). Finally, to measure the agreement between the automatic algorithms and the experts, we utilize the unweighted Cohen’s kappas.

The analysis starts from CNN model selection. Based on the literature review [2,10,38], the most recent studies: In HFUS classification [2], ultrasound IQA [38], and informative HFUS frame selection [10], favor two CNN models: DenseNet and VGG16. The most promising model will then be utilized in the following experiments. For this, we train and test both the considered architectures: DenseNet-201 and VGG16, for each expert separately (Evaluation #4). The obtained performance measures are gathered in Table 3. On this basis, we decided to select the VGG16 model for further analysis.

Since it is used in the subsequent processing steps, we first evaluated the classification performance of the selected VGG16 model for the reliable labels only (Evaluation #1). According to Cohen’s kappa analysis, we obtained Perfect Agreement (kappa = 0.9177) with the experts, and the classification accuracy equal to 0.9595. Due to the reduced (to the reliable labels) image set, these results could not be compared with any furthers. However, they proved that for the collection of images unequivocally classified by experts, the abilities of the VGG16 model for indicating the correct data are good (as we expected from [10]).

Next, we analyzed the developed extension of the direct CNN-based technique (see Figure 10). For the binary classification, the best results were obtained using path4, utilizing the CNN combination with FIS (Evaluation #6)-ACC equal to 0.9170 and f1-score equal to 0.9076. A bit worse performance measures-ACC equal to 0.9158 and f1-score equal to 0.9074, yet higher Recall-0.9266, resulted from the classical CNN-based approach-path1 (Evaluation #2). According to Cohen’s kappa analysis, both of them, as well as path2 (Evaluation #3), provided Perfect Agreement (see Table 4). The combination of three separately trained models followed by the selection of the most frequently resulted label performs worst in this case.

Finally, we evaluate the abilities of multi-class classification. By Table 1 we considered four groups and four different processing frameworks given as path5 to path8. The obtained results are collected in Figure 11. For this analysis, the best evaluation results provided the classical CNN-based version-path5, without any modification. However, same as all of the others-paths6 to path8, the Cohen’s kappa analysis indicates only Substantial Agreement. Moreover, according to the confusion matrices, the best-recognized class in all the experiments is 1 (all experts labeled the image ‘no ok’), the second is 4 (all experts labeled the image ‘ok’), and 2 i 3 are hard to distinguish by the algorithms.

## 5. Discussion and Conclusions

Since the correct acquisition of US and HFUS images is essential for further accurate data analysis, in this study, we describe possible solutions aiming at ‘correct’ image identification. We believe that this step increases the HFUS image processing reliability. The obtained results can be used twofold. First, the incorrect image data can be excluded from further automated analysis if the software classified them as incorrect. Second, the remaining data analysis can be weighted based on the system output of the kept samples. Our work is the first application in this area-HFUS images of facial skin and applying AI to this task.

The first contribution of our study is the database of 17,425 HFUS images of facial skin [47] registered by two sonographers. Two experts annotated all the image data (one annotated it twice), and a detailed analysis of this expertise is provided in this work. On this basis, we can first conclude that the proportion of correct to incorrect images decreases from 1:1.3 to 1:2 if a less experienced person performs the examination. The image analysis and classification methods would provide the worst and less reliable measurements in this case. Next, there exists a group of images, which the experts can not unambiguously annotate (see Figure 2 and Figure 3), and their automated classification by the system is also problematic. They can be considered together (labels 2 and 3), and during further numerical analysis, we can treat them as having less impact on the processing results.

**Figure 12:**
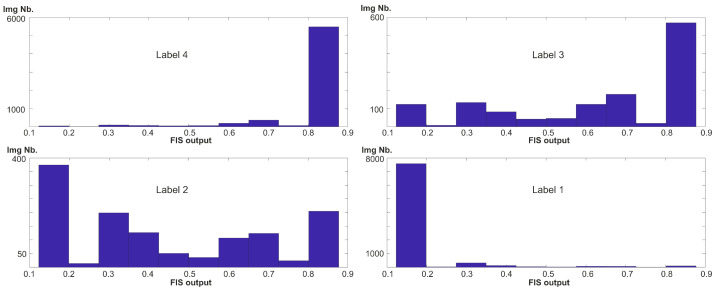
Histograms of FIS outputs obtained for the data, categorized according to the labels collected in Table 1.

The second contribution includes different developed, introduced, or just verified frameworks for automated HFUS image classification as correct-sufficient for further analysis or not. We analyzed two previously applied to similar problems [4,10], CNN models: DenseNet201 and VGG16, as having potential for HFUS frame selection. The numerical analysis benefits the latter. Using the VGG16 model as a base for further modifications, and the best among the state-of-the-art in HFUS image analysis, we proposed different frameworks to classify the image data into two or four groups. From our observation, the binary classification results are more accurate than multi-class analysis and can be applied in other HFUS image processing techniques. The best results were obtained for the developed CNN model and FIS combination. In this case the FIS-based improvement outperforms the VGG16 model. However, the limitation of the binary solutions is that they are trained and verified using the labels resulting from the voting step. It means that the ‘correct’ group includes the image data labeled as ‘ok’ both by all the experts and only two of them. The same problem appears for the ‘incorrect’ group. This solution assumes that the data annotated as ‘ok’ by most of the experts can be considered in the other processing steps (i.e., segmentation or further classification). To reduce the influence of two middle labels (2 and 3) on image analysis, we suggest assigning the confidence level to each analyzed image, utilizing the FIS outputs. The histograms of FIS outputs for binary classification are given in Figure 12. It is worth mentioning that both the analyzed models, as well as the FIS systems, are made available in [47].

To reduce the imbalance of group size, especially in four-class analysis, it is possible to introduce the augmentation step during training four-class. However, based on our previous experiences, the augmentation procedures should be selected carefully to avoid additionally produced artifacts due to the specific data appearance. Besides of this, future improvement can include three-class analysis, other body parts and diseases, and a broader range of frequencies and HFUS machines commonly used in dermatological practice, like 33, 50, or 75 MHz. Additionally, we plan to introduce FIS output weights as the pre-processing step for previously described segmentation [17] and classification [4] frameworks to evaluate their influence on the obtained results. Moreover, it needs to be validated in clinical practice.

In conclusion, this study describes the first step of the HFUS image analysis. The developed algorithm makes it possible to automatically select correctly acquired US frames among all the images collected during the US examination. This method applied as the pre-processing step will decrease the influence of misclassifications or over/under segmentations and improve the reliability of the measurements. Furthermore, it can be used instead of pre-processing steps targeting artifact reduction. The frame selection step is crucial since the proportion of correct to incorrect scans is about 1.5. On the other hand, due to the high amount of images acquired during the single examination, manual data selection is time and cost-consuming, and the developed technique solves this problem.

## Figures and Tables

**Table 1:** The size of individual groups.

Group Label	Description	Size
1	all experts labeled the image ‘no ok’	8398
2	one expert labeled the image ‘no ok’	1261
3	two experts labeled the image ‘no ok’	1324
4	all experts labeled the image ‘ok’	6442

**Table 2:** The database information.

ID	8032021	15022021	12042021	7062021
nb. of patients	43	43	40	40
nb. of images	4385	5840	4384	2816

**Table 3:** Performances of CNN models.

		ACC	Precision	Recall	f1-Score
Expert1:	DenseNet-201	0.8790	0.8440	0.8723	0.8579
	VGG16	**0.8982**	**0.8738**	**0.8849**	**0.8793**
Expert2:	DenseNet-201	0.8682	0.8322	0.8644	0.8480
	VGG16	**0.8907**	**0.8713**	**0.8718**	**0.8716**
Expert3:	DenseNet-201	0.8802	0.8632	0.8974	0.8800
	VGG16	**0.8999**	**0.8855**	**0.9135**	**0.8993**

**Table 4:** Experts and algorithms agreement-unweighted Cohen’s kappa.

	Kappa	Agreement		Kappa	Agreement
Evaluation#1	0.9177	Perfect	Evaluation#7	**0.7193**	Substantial
Evaluation#2	0.8302	Perfect	Evaluation#8	0.6855	Substantial
Evaluation#3	0.8214	Perfect	Evaluation#9	0.6808	Substantial
Evaluation#5	0.7822	Substantial	Evaluation#10	0.6730	Substantial
Evaluation#6	**0.8322**	Perfect			

## Data Availability

The dataset is available as: Czajkowska, J.; Juszczyk. J.; Piejko, L.; Glenc-Ambroży, M. (2022), High-Frequency Dataset of Facial Skin, Mendeley Data, V1, doi:10.17632/ td8r3ty79b.1.
